# Reference indices for evaluating kidney dimensions in children using anthropometric measurements

**DOI:** 10.4102/sajr.v24i1.1882

**Published:** 2020-08-05

**Authors:** Salome N. Ezeofor, Godson E. Anyanwu, Emmanuel N. Obikili

**Affiliations:** 1 Department of Radiation Medicine, Faculty of Medical Sciences, College of Medicine, University of Nigeria, Ituku Ozalla, Enugu Nigeria; 2 Department of Anatomy, Faculty of Basic Medical Sciences, College of Medicine, University of Nigeria, Enugu Nigeria

**Keywords:** Kidney, Renal sizes, Children, Ultrasound, Nigeria

## Abstract

**Background:**

Kidney pathologies often result in change in renal size. Knowledge of normal kidney sizes is important for screening, diagnosis, prognosis and follow-up management of paediatric renal diseases.

**Objectives:**

The aim of this study was to establish the age-, height- and weight-matched kidney dimensions in apparently healthy Nigerian children.

**Method:**

A descriptive, cross-sectional study of right and left kidney parameters (length, width, thickness and volume) of 1315 school-aged Nigerian children was conducted over 8 months. Ages ranged from 5 to 17 years. Parameters were obtained using a General Electric (GE) LOGIC 400CL ultrasound machine. Kidney dimensions were correlated with age, sex and anthropometric measurements.

**Results:**

Normative values for all the kidney parameters for each age, height and weight groups and also gender were established for the study population. The left kidneys were noted to be longer and thicker, and of more volume than the right kidneys. The right kidneys were seen to be wider (*p* < 0.01). Length of the left kidneys in females was noted to be more than those of the males in the age- and weight-matched categories (*p* < 0.05). The width of both kidneys was higher in the males in all the categories (*p* < 0.05). Males showed higher values of thickness and volume in the height category. All the renal parameters significantly correlated with body size indicators, except for body mass index.

**Conclusion:**

This study has established gender-, age-, weight- and height-specific range of values of the kidney parameters of apparently healthy children together with regression models.

## Introduction

Anomalies of renal sizes are associated with and are manifestations of diseases involving the kidneys.^1^ The importance of accurate reference values of children’s renal sizes measured by ultrasonography cannot be overemphasised.^2^ Ultrasonography is without risk of ionising radiation and is therefore safe in the evaluation of growing children. It also provides a quick and accurate assessment of other visceral organ dimensions.^3^ Reports have demonstrated that renal length differs from race to race.^4,5,6^ The size and weight of an organ have also been shown to be influenced by environmental variations, ethnicity, hereditary components, routine diet, water intake^7,8^ and high altitudes, where atmospheric pressure is reduced, with the partial pressure of oxygen altering the physiology of the kidneys.^9^

The kidneys can be affected by congenital or acquired diseases, either localised or systemic. Examples are solitary kidney, renal hypoplasia,^10^ multicystic and polycystic kidneys, acute malaria because of plasmodium falciparum,^11^ auto-immune diseases such as Kawasaki disease,^12^ recurrent urinary tract infection, neoplasms, urolithiasis, trauma, drugs, ingestion of native concoctions, diabetes, hypertension, renal artery stenosis and so on. Knowledge of renal size helps in differentiating acute from chronic kidney diseases (CKD).^2,13^ Chronic diseases include diseases that reduce the size of the kidneys such as chronic glomerulonephritis, nephrosclerosis and diabetic nephropathy, and those that increase its sizes such as multi- or polycystic kidney diseases and so on. In addition, renal length and volume are very important parameters for numerous purposes such as the assessment of candidates for/with kidney transplant, decision in obtaining renal biopsies and follow-up of patients with end-stage liver disease in which nephromegaly and increased echogenicity of renal cortex can be associated with pathological findings (renal size usually reverses after liver transplant). Kidney size is an important parameter used for the clinical evaluation of renal abnormalities, such as atrophy, hypoplasia and hypertrophy in children. Sonography is used to monitor the kidneys of children before and after liver transplants with sizes compared with published normative values.^14^ Renal involvement can be a part of a syndrome such as Beckwith–Wiedemann syndrome (BWS). This syndrome is reported to have a high risk of development of embryonic tumours such as Wilm’s tumour.^15^ Screening protocols with ultrasonography have been implemented in some countries for the early detection of these tumours^16^ because a criterion for its diagnosis is evidence of renal enlargement.

A study by Jones et al.^17^ demonstrated that renal volume, which correlates better with renal mass, is a more sensitive means of diagnosing kidney abnormality than any single linear measurement. Also at autopsy, renal volume has been reported to correlate well but indirectly with the number of functioning nephrons,^18^ hence its inclusion in this study.

A Nigerian study on paediatric hospital admissions by Esezobar et al.^19^ showed that acute renal disease accounted for up to 82.9% of admissions. It is invaluable to have a more comprehensive, standardised, sonographic measurement for use in the course of the renal assessment of a child, hence this study.

## Materials and methods

This was a descriptive, cross-sectional study of the kidney parameters of apparently healthy, school-aged, Nigerian children without any known renal disease. Whilst informed consent was received from the parents of the children, child assent was also obtained from each child involved in this study. Detailed medical history of each child was acquired from the parents including pre-existing diseases that could affect the kidneys, past urologic surgeries and known history of chronic diseases. After clinical examination of each child by one of the authors who is a clinician, only apparently healthy children were recruited for the study.

Clinical exclusion criteria were fever, periorbital or pedal edema, macular or maculopapular rashes and sickle cell anaemia. Imaging exclusion criteria were altered echogenicity, presence of renal cysts, urolithiasis, unilateral kidney, hydronephrosis, ureterocele, renal ectopia, horseshoe kidneys and other developmental anomalies, and neoplasm.

A total of 1315 children (633 boys and 682 girls) between the ages of 5 and 17 years were selected for this study using a random selection method. Age, sex and anthropometric measurement of the body size indicators such as weight (WT), height (HT), body surface area (BSA) and body mass index (BMI) were obtained for each subject. Using the vertical scale of a portable stadiometer, each participant was placed, without shoes, in an upright position with the head held in the Frankfort plane and the height measured to the nearest 0.5 cm. With each participant lightly clothed, weight was measured with a weighing scale to the nearest 0.1 kg.

**FIGURE 1:**
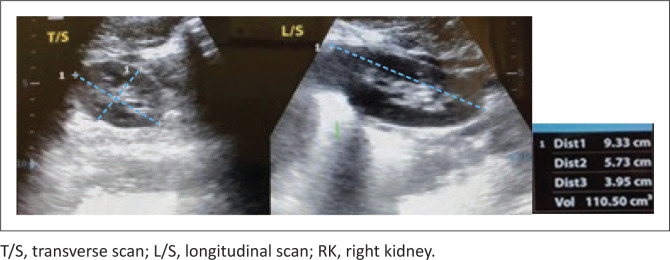
Showing points of measurements of a kidney.

Kidney parameters were obtained using a GE LOGIC 400CL ultrasound machine made by GE medical systems with a 3.5 MHz curvilinear probe. Renal sizes by ultrasound were obtained by one of the authors who is a radiologist. To achieve greater accuracy, two sequential measurements were taken and the mean calculated. Kidney measurements were obtained with subjects in a prone position^20^ and in quiet respiration. The bipolar length of each kidney was measured from the highest to its lowest point. The width and thickness were obtained in the transverse plane in an orthogonal direction, near the renal hilum but free of the pelvis. The renal thickness or anterioposterior (AP) diameter, was measured in the same transverse plane with a line perpendicular to the width (at its central highest point), as shown in Figure 1. The probe therefore was not exactly perpendicular to the skin. No subject was included more than once. No sedation nor any preparation was used. The mean renal length and 5th and 95th percentiles were determined for each age. The BSA and BMI were calculated using the respective formulas:

BSA = (weight (kg) × height (m)/3600)^1/2^ (Mosteller formula).

BMI = weight (kg)/height^2^ (m).

Renal volume = length (cm) × width (cm) × thickness (cm) × 0.523.

### Ethical considerations

Ethical approval to conduct the study was obtained from the College of Medicine Research Ethics Committee, University of Nigeria Enugu Campus (Reference no. 070/06/2019).

## Results

The mean values of the left and right renal dimensions of the various age groups of the studied population have been presented in Table 1. Table 2 presents a height-matched comparison, whilst Table 3 shows a weight-matched comparison. Table 4 presents the renal correlation. The regression formula for the various renal dimensions is presented in Table 5.

**TABLE 1:** Distribution of mean renal dimensions (cm) based on various age categories (years).

Parameters	Sex	6 years	7 years	8 years	9 years	10 years	11 years	12 years	13 years	14 years	15 years	16 years	17 years
Right kidney length	M	8.2 ± 0.6	8.6 ± 0.6	8.5 ± 0.6	8.9 ± 0.8	8.9 ± 0.7	9.0 ± 0.7	9.0 ± 0.9	9.7 ± 0.7	10.0 ± 0.8	10.2 ± 0.7	10.4 ± 0.9	10.1 ± 0.8
	F	8.08 ± 0.6	8.5 ± 0.6	8.6 ± 0.9	8.8 ± 0.7	9.0 ± 0.8	9.0 ± 0.9	9.6 ± 0.7	9.5 ± 1.8	10.0 ± 1	10.0 ± 0.7	10.6 ± 1	10.2 ± 0.7
Left kidney length	M	8.3 ± 0.6	8.6 ± 0.8	8.6 ± 0.7	9.0 ± 0.7	8.8 ± 1.1	9.1 ± 0.8	9.3 ± 1.0	9.4 ± 1.9	10.2 ± 0.8	10.6 ± 1.0	10.6 ± 0.8	10.3 ± 0.8
	F	8.4 ± 0.7	8.5 ± 0.6	8.7 ± 0.6	9.0 ± 0.8	9.1 ± 0.9	9.1 ± 1	9.7 ± 0.8	10.0 ± 1.0	10.5 ± 0.9	10.5 ± 0.8	10.9 ± 0.9	10.2 ± 0.9
Right kidney width	M	5.7 ± 0.7	6.1 ± 0.5	6.1 ± 0.6	6.3 ± 0.8	6.3 ± 0.6	6.4 ± 0.5	6.5 ± 0.8	7.1 ± 0.7	7.3 ± 0.7	7.2 ± 0.7	7.3 ± 0.6	7.3 ± 0.7
	F	5.7 ± 0.6	6.0 ± 0.5	6.2 ± 0.7	6.2 ± 0.6	6.3 ± 0.6	6.3 ± 0.6	6.8 ± 0.7	6.9 ± 0.8	7.1 ± 0.7	7.1 ± 0.6	7.4 ± 0.8	7.5 ± 0.8
Left kidney width	M	5.7 ± 0.5	6.2 ± 0.5	6.2 ± 0.5	6.3 ± 0.6	6.3 ± 0.6	6.3 ± 0.5	6.4 ± 0.8	7.0 ± 0.7	7.4 ± 0.7	7.2 ± 0.8	7.3 ± 0.6	7.5 ± 0.7
	F	5.7 ± 0.6	6.0 ± 0.4	6.1 ± 0.6	6.1 ± 0.6	6.3 ± 0.6	6.2 ± 0.7	6.8 ± 0.7	7.1 ± 0.6	7.1 ± 0.6	7.3 ± 0.5	7.4 ± 0.7	7.3 ± 0.6
Right kidney thickness	M	3.2 ± 0.4	3.4 ± 0.3	3.4 ± 0.4	3.5 ± 0.4	3.5 ± 0.4	3.6 ± 0.3	3.7 ± 0.6	3.8 ± 0.6	4.0 ± 0.5	4.0 ± 0.5	4.1 ± 0.4	4.0 ± 0.3
	F	3.1 ± 0.4	3.4 ± 0.4	3.3 ± 0.4	3.5 ± 0.4	3.6 ± 0.4	3.6 ± 0.5	3.8 ± 0.0.8	3.9 ± 0.4	4.0 ± 0.4	4.7 ± 0.5	4.1 ± 0.5	4.0 ± 0.5
Left kidney thickness	M	3.7 ± 0.5	4.0 ± 0.4	4.0 ± 0.4	4.2 ± 0.5	4.1 ± 0.7	4.1 ± 0.3	4.1 ± 0.5	4.5 ± 0.7	4.7 ± 0.6	4.7 ± 0.6	4.7 ± 0.5	4.9 ± 0.4
	F	3.7 ± 0.4	3.8 ± 0.4	3.9 ± 0.4	4.0 ± 0.5	4.2 ± 0.5	4.1 ± 0.6	4.6 ± 0.6	4.5 ± 0.5	4.6 ± 0.6	4.7 ± 0.6	4.8 ± 0.5	4.8 ± 0.6
Right kidney volume	M	75.8 ± 20.1	88.3 ± 16.7	90.2 ± 19.3	100.3 ± 20.4	98.2 ± 22.0	103.3 ± 19.5	110.5 ± 36.7	132.5 ± 31.1	148.7 ± 32.8	148.9 ± 32.0	155.9 ± 30.1	146.7 ± 17.5
	F	72.9 ± 15.2	85.5 ± 17.0	90.6 ± 21.8	95.5 ± 20.1	102.4 ± 23.7	103.5 ± 27.7	124.7 ± 32.6	131.3 ± 32.6	144.6 ± 34.1	168.0 ± 34.3	164.4 ± 47.5	152.6 ± 28.4
Left kidney volume	M	89.2 ± 20.6	106.4 ± 21.4	106.7 ± 23.4	119.1 ± 25.8	111.7 ± 27.8	116.7 ± 22.0	127.1 ± 38.7	146.8 ± 45.4	178.2 ± 40.3	181.2 ± 51.4	188.8 ± 37.0	190.7 ± 41.5
	F	88.4 ± 15.4	97.4 ± 14.2	104.2 ± 22.5	111.8 ± 29.0	120.5 ± 28.2	118.6 ± 35.7	151.4 ± 38.3	160.9 ± 40.5	173.8 ± 41.3	176.5 ± 34.8	195.1 ± 44.1	182.5 ± 43.2

M, male; F, female.

**TABLE 2:** Distribution on mean renal dimensions (cm) based on various height categories (cm).

Parameters	Sex	≤ 105 cm	106–110 cm	111–115 cm	116–120 cm	121–125 cm	126–130 cm	131–135 cm	136–140 cm	141–145 cm	146–150 cm	≥ 151 cm
Right kidney length	M	7.20	7.58	7.94	8.08	8.39	8.59	8.73	8.80	9.00	9.09	9.65
	F	6.90	7.69	7.93	8.02	8.21	8.48	8.73	8.79	8.95	9.14	9.69
Left kidney length	M	7.17	7.67	8.13	8.23	8.44	8.52	8.78	8.85	9.08	9.08	9.69
	F	7.97	7.89	8.21	8.32	8.35	8.61	8.78	9.01	9.05	9.34	9.63
Right kidney width	M	5.00	5.11	5.45	5.64	6.08	6.11	6.15	6.26	6.41	6.60	6.92
	F	5.47	5.39	5.49	5.71	5.77	6.09	6.14	6.23	6.48	6.36	6.71
Left kidney width	M	4.53	5.36	5.60	5.64	6.05	6.06	6.20	6.29	6.32	6.43	6.82
	F	5.23	5.15	5.40	5.69	5.80	5.90	6.05	6.14	6.33	6.33	6.75
Right kidney thickness	M	2.70	3.06	3.10	3.17	3.37	3.43	3.41	6.26	3.59	3.74	3.73
	F	3.13	2.95	3.11	3.22	3.19	3.31	3.39	3.50	3.60	3.62	3.85
Left kidney thickness	M	2.83	3.58	3.52	3.75	3.88	4.10	4.04	6.29	4.13	4.24	4.42
	F	3.33	3.50	3.61	3.65	3.80	3.84	3.91	4.03	4.22	4.26	4.54
Right kidney volume	M	48.54	60.30	67.43	73.16	86.96	90.46	92.35	96.54	104.04	113.25	125.48
	F	59.28	61.35	68.33	74.20	76.02	86.24	91.50	96.58	104.89	106.72	128.01
Left kidney volume	M	46.71	72.75	80.46	87.09	100.00	105.41	110.70	113.57	119.34	125.34	148.18
	F	69.82	71.40	80.60	86.16	92.14	97.70	104.68	112.58	121.94	127.54	149.79

M, male; F, female.

**TABLE 3:** Distribution of mean renal dimensions (cm) based on various weight categories (kg).

Parameters	Sex	15–20 kg	21–25 kg	26–30 kg	31–35 kg	36–40 kg	41–45 kg	46–50 kg	51–55 kg	56–60 kg	61–65 kg	66–70 kg	≥ 70 kg
Right kidney length	M	7.66	8.22	8.67	8.87	9.18	9.47	9.95	10.15	9.96	10.27	10.74	10.64
	F	7.78	8.20	8.69	8.84	9.07	9.65	9.53	10.02	10.41	10.61	10.59	10.47
Left kidney length	M	7.81	8.29	8.67	8.93	9.36	9.60	10.02	10.11	10.04	10.47	10.98	10.84
	F	8.13	8.40	8.77	8.97	9.28	9.86	9.86	10.46	10.52	10.74	11.09	10.67
Right kidney width	M	5.37	5.77	6.13	6.31	6.63	6.83	6.94	7.11	7.25	7.39	7.66	7.43
	F	5.44	5.82	6.10	6.29	6.50	6.79	6.76	7.09	7.25	7.46	7.47	7.68
Left kidney width	M	5.46	5.79	6.11	6.26	6.64	6.70	6.83	7.09	7.23	7.44	7.47	7.63
	F	5.34	5.77	5.98	6.20	6.35	6.87	6.83	7.24	7.14	7.46	7.54	7.83
Right kidney thickness	M	3.01	3.21	3.42	3.53	3.66	3.82	3.91	3.98	4.02	4.06	4.11	4.16
	F	3.05	3.21	3.34	3.53	3.56	3.88	3.93	3.97	4.06	5.60	4.30	4.40
Left kidney thickness	M	3.49	3.75	4.02	4.10	4.19	4.37	4.43	4.64	4.70	4.89	5.01	4.93
	F	3.55	3.75	3.90	4.03	4.25	4.48	4.55	4.76	4.61	4.72	4.95	5.12
Right kidney volume	M	62.67	77.07	91.29	103.74	116.46	130.18	142.17	151.41	152.92	161.63	176.79	171.72
	F	65.08	77.18	89.01	98.50	105.87	127.66	129.50	141.66	154.79	217.68	172.29	178.05
Left kidney volume	M	74.76	90.67	106.49	121.02	137.67	148.62	160.71	174.88	179.40	199.96	216.94	215.32
	F	77.60	91.23	103.00	112.76	126.34	153.32	154.21	180.87	174.93	189.83	209.24	215.08

M, male; F, female.

**TABLE 4:** Correlation matrix coefficients of renal dimensions with age and body size indicators.

Parameters	Age	Height	Weight	Body Mass Index	Body Surface Area	Right Kidney Length	Right Kidney Thickness	Right Kidney Width	Right Kidney Volume	Left Kidney Length	Left Kidney Thickness	Left Kidney Width	Left Kidney Volume
Age	1	0.371**	0.747**	0.052	0.785**	0.583**	0.289**	0.568**	0.503**	0.565**	0.473**	0.578**	0.617**
Height	0.371**	1	0.413**	−0.101**	0.955**	0.331**	0.158**	0.280**	0.267**	0.315**	0.263**	0.313**	0.339**
Weight	0.747**	0.413**	1	0.049	0.979**	0.709**	0.388**	0.671**	0.646**	0.690**	0.626**	0.694**	0.790**
Body Mass Index	0.052	−0.101**	0.049	1	−0.061*	0.051	0.010	0.069*	0.040	0.054	−0.002	0.043	0.033
Body Surface Area	0.785**	0.955**	0.979**	−0.061*	1	0.719**	0.384**	0.676**	0.639**	0.701**	0.628**	0.699**	0.788**
Right Kidney Length	0.583**	0.331**	0.709**	0.051	0.719**	1	0.323**	0.642**	0.658**	0.715**	0.513**	0.633**	0.714**
Right Kidney Thickness	0.289**	0.158**	0.388**	0.010	0.384**	0.323**	1	0.373**	0.890**	0.317**	0.338**	0.360**	0.396**
Right Kidney Width	0.568**	0.280**	0.671**	0.069*	0.676**	0.642**	0.373**	1	0.693**	0.571**	0.562**	0.679**	0.698**
Right Kidney Volume	0.503**	0.267**	0.646**	0.040	0.639**	0.658**	0.890**	0.693**	1	0.566**	0.516**	0.598**	0.659**
Left Kidney Length	0.565**	0.315**	0.690**	0.054	0.701**	0.715**	0.317**	0.571**	0.566**	1	0.465**	0.647**	0.817**
Left Kidney Thickness	0.473**	0.263**	0.626**	−0.002	0.628**	0.513**	0.338**	0.562**	0.516**	0.465**	1	0.642**	0.820**
Left Kidney Width	0.578**	0.313**	0.694**	0.043	0.699**	0.633**	0.360**	0.679**	0.598**	0.647**	0.642**	1	0.885**
Left Kidney Volume	0.617**	0.339**	0.790**	0.033	0.788**	0.714**	0.396**	0.698**	0.659**	0.817**	0.820**	0.885**	1

* , *p* < 0.05;

** , *p* < 0.01.

**TABLE 5:** Distribution of the regression formula for the various renal dimensions using age and body size indicators.

Dependent variable	Regression formula	*p*-value
Right Kidney Length	(5.91) + Age (0.04) + HT (0.01) +WT (0.03)	< 0.0001
Right Kidney Thickness	(2.43) + Age (0.004) + HT (0.002) + WT (0.02)	< 0.0001
Right Kidney Width	(4.48) + Age (0.04) + HT (0.004) + WT (0.02)	< 0.0001
Left Kidney Length	(5.58) + Age (0.05) + HT (0.01) + WT (0.021)	< 0.0001
Left Kidney Thickness	(2.68) ‒ Age (0.004) + HT (0.005) + WT (0.03)	< 0.0001
Left Kidney Width	(4.23) + Age (0.043) + HT (0.005) + WT (0.03)	< 0.0001
Right Kidney Volume	(30.67) + Age (0.64) – HT (0.003) + WT (2.06)	< 0.0001
Left Kidney Volume	(33.91) + Age (0.079) + HT (0.02) + WT (2.39)	< 0.0001

Significant asymmetry was noted in all the measured renal parameters. Whilst the left renal length, thickness and volume were greater than those of the right in all the age-, weight- and height-matched categories (*p* < 0.01), the reverse was noted for the right renal width. Significant sexual dimorphism was observed in the kidney dimensions (*p* < 0.05). The left kidney was significantly longer in females (*p* < 0.05) in the age and weight categories, whilst the males showed significantly wider kidneys in all the categories (age, height and weight). Females were also noted to have thicker kidneys in most of the categories (age and weight) and larger volume in all the categories than males, though without statistical significance (*p* > 0.05).

Of those who did not meet the imaging inclusion criteria, six subjects were found to have some congenital renal pathologies. Two of these six children had unilateral kidneys, one subject had a left ureterocele with gross left-sided hydronephrosis whilst the three remaining subjects had ectopic kidneys visualised in the pelvis. These abnormal findings were communicated to the parents of each of these children. The final study sample size was 1315.

## Discussion

Several studies on renal sizes have been reported in neonates/infants,^15,21,22,23^ children,^14,24,25,26,27,28^ adults^4,6,29,30,31^ and geriatric subjects.^5,32^ This study shows that the left kidney is longer, thicker and more voluminous than the right kidney. This is consistent with previous reports^22,26,33,34^ but contrary to research that noted that there is no statistical difference between the left and right kidneys.^20,35^

Sexual dimorphism was observed in our study – the left kidney in females was longer than the left kidney of males when age- and weight-matched. This is in agreement with studies conducted in New York by Chen et al.^24^ In addition, sexual dimorphism was noted in another study in infants.^22^ However, some other researchers showed no sexual dimorphism in their reports, although some commented that the rate of general somatic growth and body proportion are different between boys and girls.^2,25,26,28,33,36^

Studies carried out on Indian and Chinese children^25,37^ showed a progressive increase in renal length and volume with age. This increase with age was not consistent in our study population until 9 years of age and beyond. The difference in the number of participants within each of the groups and/or variations in the nutritional levels of the children in these particular age groups may be contributory factors to this observation.

The renal parameters in our study correlated best with BSA and weight *p* < 0.05. This is in agreement with some reports^38,39^ but contrary to other studies^10,34,36,38,40,41^ in which there was best correlation with height. Yet, other researchers have reported correlation with both height and weight.^28,42,43,44^ Oh et al.^2^ observed that height is the most influencing factor amongst the somatic variables in children < 2 years of age, whilst weight and age have good correlation with renal length from 2 to 12 years of age. A study by Pantoja et al.^42^ also noted that the kidneys were significantly larger in the obese subjects than in children with normal weights. Previous reports in low birth weight infants and premature deliveries have observed that these children have low nephron number and therefore reduced renal volumes^45,46,47^ and discovered they have a related risk of hypertension and renal diseases.^45^ One can speculate that the bigger the body size of a person, the higher the nephron number to take care of the body’s metabolic needs.

Previous Nigerian-based results on renal parameters of adults^30,48^ demonstrated that the renal parameters correlated best with weight compared with the current study where BSA had the strongest correlation followed by weight. We have demonstrated that age and all the body size indicators significantly correlate (*p* < 0.01) with all the renal dimensions with the exception of BMI which is in agreement with reports by Younus et al.^28^ that BMI may not be a good predictor of renal measurement. However, this is contrary to another report where BMI was demonstrated to significantly relate to renal length.^42^

The prevalence of congenital anomaly as revealed in this study was 0.46% which is slightly lower than the 0.89% recorded by Scott et al.^22^ in a similar study carried out in infants.

## Conclusion

We have established age-, weight- and height-specific normal values of the kidneys in apparently healthy Nigerian children and developed regression equations for adequate evaluation and follow-up of renal diseases in clinical radiology and general medicine. We also noted significant sexual dimorphism and bilateral asymmetries in the kidney parameters of the studied population.

## Limitation of the study

Urinalysis, serum electrolyte, urea and creatinine or glomerular filtration rate tests were not carried out for the study population, which may have further eliminated possibilities of including children with renal diseases. These investigations are more specific for kidney function than they are for renal morphology, which is the focus of this work.
